# Supplementary material for: Polyamine biosynthesis dysregulation in Alzheimer’s disease and Down syndrome cellular models
Source: bioRxiv. 2025 Feb 2:2025.01.31.635912. Preprint. [Version 2] doi: 10.1101/2025.01.31.635912 (PMC11838436; doi:10.1101/2025.01.31.635912)
Supplement: 1 [file NIHPP2025.01.31.635912V2-supplement-1.pdf]

bioRxiv preprint doi: https://doi.org/10.1101/2025.01.31.635912; this version posted February 3, 2025. The copyright holder for this preprint (which was not certified by peer review) is the author/funder, who has granted bioRxiv a license to display the preprint in perpetuity. It is made available under aCC-BY 4.0 International license.

SUPPLEMENTARY TABLES

Supplemental Table 1. *Antibodies utilized in immunohistochemistry (IHC) and Western blot (WB) experiments at indicated concentrations according to manufacturer's recommendations for specific applications.*

Antibody	Manufacturer	Application	Concentration
Hoechst stain	Sigma (14530)	IHC	1 ug/mL in PBS
MOAB-2	Abcam (ab126649)	IHC	5 ug/mL in primary solution
B-Amyloid peptide	Aves (ABN)	ICC	1:2000 in blocking
APP A4	EMD Millipore, MAB348	IHC	5 ug/mL in primary solution
ODC	Abcam (ab97395)	WB	1:1000 in blocking
ODC	Abcam (193338)	IHC	1:1000 in blocking
BACE1	Abcam (183612)	WB	1:1000 in blocking
Spermidine synthase	Abcam (241496)	WB	1:1000 in blocking
Spermine synthase	Abcam (156879)	WB	1:1000 in blocking
Arginase 1	Cell Signaling (93668)	WB	1:1000 in blocking
SAT1	ThermoFisher (PA1-16992)	WB	1:1000 in blocking
B-actin	Abcam (ab8227)	WB	1:1000 in blocking
Anti-mouse HRP	BioRad (1706516)	WB	1:500 in PBS-T
Anti-rabbit HRP	BioRad (1706515)	WB	1:500 in PBS-T

bioRxiv preprint doi: https://doi.org/10.1101/2025.01.31.635912; this version posted February 3, 2025. The copyright holder for this preprint (which was not certified by peer review) is the author/funder, who has granted bioRxiv a license to display the preprint in perpetuity. It is made available under aCC-BY 4.0 International license.

Supplemental Table 1. *The hippocampal brain tissue used was obtained from a collaboration with Dr. Ann-Charlotte Granholm and the MUSC Carroll Campbell Jr. Neuropathology laboratory. This South Carolina Brain Bank contains frozen and fixed tissues from >250 Alzheimer cases and Controls, and all cases have undergone neuropathological and clinical staging including the ABC assessment as described by Jack et al [50] and recently amended by DeTure and Dickson [51] and Aldecoa et al., [10]*

Case Year	Case Num	Age at Death	Sex	Ethnicity	PMI	Last Clinical Dx	Primary Neuropath (AD) Dx	Secondary Neuropath Dx(s)	BRAAK Tangle Stage	CERAD Plaque Stage	Thal Phase	ABC Score	ADNC	Notes	MF (g)
2015	32	49	M	White	6.03	Dem	Alzheimer's disease (Clinical history of DS)		Stage 6	Stage C	Phase 3	A2B3C3	High		0.49
2017	7	47	F	White	6.5	Dem	Alzheimer's disease (Clinical history of DS)		Stage 6	Stage C	Phase 3	A2B3C3	High		0.66
2019	58	63	F	White	3.87	Dem	Alzheimer's disease (Clinical history of DS)	Vascular Dementia (other)	Stage 6	Stage C	Phase 4	A3B3C3	High		0.47
2015	32	49	M	White	6.03	Dem	Alzheimer's disease (Clinical history of DS)		Stage 6	Stage C	Phase 3	A2B3C3	High		0.49
2017	3	57	M	White	4.25	Dem	Alzheimer's disease (Clinical history of DS)		Stage 6	Stage C	Phase 5	A3B3C3	High		0.48
2000	30	61	M		10.5		Alzheimer's disease (Clinical history of DS)		Stage 6	Stage C				no NACC	0.49
